# Genetic content of Influenza H3N2 vaccine seeds

**DOI:** 10.1371/currents.RRN1165

**Published:** 2010-09-05

**Authors:** Corinne Bergeron, Martine Valette, Bruno Lina, Michele Ottmann

**Affiliations:** ^*^Université Lyon 1; ^†^CNR Virus Influenza région sud - Hospices Civils de Lyon; ^‡^NIC Lyon & UCBL-CNRS FRE 3011, Lyon, France and ^§^VirPath FRE3011, Université Lyon1, France

## Abstract

Influenza vaccine seeds produced in chicken eggs are selected through HA and NA surface glycoproteins antigenicity, as well as through high replicative ability. Here we characterize the genetic content of recently used thirteen H3N2 influenza vaccine seeds. Interestingly, sequence analysis of the vaccine seeds shows reassortment events leading to PR8:H3N2 segment constellations, ranging from the 6:2 to 2:6 constellations. This study shows that the H3N2 PB1 is the most frequent internal segment incorporated in the tested vaccines seeds.

****

## **Introduction**

Influenza A viruses are members of the *Orthomyxoviridae* family [1] . Their genomes consist of eightsingle-stranded RNA segments of negative polarity [2] . The influenza genome evolves in two ways: antigenic shift and antigenic drift. Antigenic shift involves a genetic rearrangement of segments of different viral origins during a co-infection event and might result in the emergence of pandemic virus [3] . Antigenic drifts are causedby frequent mutations during the replication step introduced by the viral polymerase complex: the viruses can be antigenically different and cause epidemics [4]. 

Vaccination is the cornerstone of influenza prevention, and constant surveillance is necessary as a result of changes in the virus driven by antigenic drift. Vaccines are protective if the strains composing them antigenically match circulating strains. The World health organization (WHO) coordinates influenza surveillance programs and twice a year, reference strains included in vaccines of the North and South hemispheres are sent to manufacturers. Influenza vaccines are usually trivalent, including representative surface antigens of the three major subtypes circulating in humans: A(H1N1), A(H3N2) and B. Epidemic vaccines are prepared almost entirely from viruses grown in the allantoic cavity of 9- to 11-days-old embryonated chicken eggs [5] . Each type A vaccine seed is produced by co-infection of chicken eggs with one selected reference strain (A(H1N1) or A(H3N2)) and with the acceptor strain, A/Puerto Rico/8/1934 (H1N1) (PR8), an attenuated human strain with high growth performance. Vaccine seeds are selected on two criteria: high growth properties and presence of reference strain major antigens, i.e. hemagglutinin (HA) and neuraminidase (NA) proteins. However, vaccine production in chicken eggs has several disadvantages given that the method is time comsuming and cumbersome. In recent years, several manufacturers have explored alternative approaches using accredited mammalian cell lines, e.g. African Green monkey kidney (Vero) cells [6] , Madin-Darby canine kidney (MDCK) cells [7] or PER.C6 cells [8] . In the future, plasmid-based reverse genetics could be applied for the production of viruses from selected plasmids (for reviews see [9] and [10] ). 

In this study, we analyzed the segment constellation of thirteen H3N2-based vaccine seeds in order to define their exact genetic content. Our results reveal that H3N2 internal segments were present in the vaccine seeds more han expected. 

## **Methods: Vaccine seed sequencing**

Vaccine seeds from manufacturers were kindly provided by the Centre National de Référence Grippe France Sud. Thirteen H3N2 vaccine seeds were analyzed (Table 1). Viral RNAs (vRNA) were extracted directly from virus-containing allantoic fluid (150 µl) using RNA isolation kit (Macherey Nagel) according to the manufacturer’s instructions. vRNAs were eluted in 50 µl of nuclease-free water. A reverse-transcription step was performed with an Uni12 primer (5’-AGCGAAAGCAGG-3’) [11] and 10 µl of extracted vRNA, in a total volume of 20 µl for 5 min at 70°C, followed by 1 h at 42°C and 5 min at 65°C. Fragments of each of the eight viral cDNA were generated from 2 µl of cDNA using GoTaq DNA polymerase (Promega) and the following PCR cycling conditions: 2 min at 94°C, followed by 39 cycles of amplification (30 sec at 94°C, 1 min at 57 or 58°C, and 2 min at 72°C) and a final extension step of 3 min at 72°C. Primer sequences are available on request. All PCR products were verified by agarose gel electrophoresis and purified using NucleoSpin Extract II (Macherey Nagel). After purification, sequencing was performed by Eurofins MWG Operon or GATC Biotech. 

The sequences of the internal segments of vaccine seeds were compared with the corresponding PR8 or H3N2 reference strain sequences as available in Influenza Sequence Database [12]. The nucleotide sequences obtained in this study were available from GenBank under accession numbers CY045983 to CY046060 [13].  

## **Results: Sequence analysis of vaccine seeds**

Thirteen of the manufacturer vaccine seeds used between the 1996-1997 and 2007-2008 seasons were sequenced to detail their gene constellation (Table 1). The sequences of glycoproteins-encoding segments were not analyzed, since the presence of H3N2 antigens (HA and NA) in these seeds was a selection criteria. The sequences of the internal segments (PB1, PB2, PA, NP, M and NS) of vaccine seeds were compared with the corresponding PR8 or H3N2 reference strain sequences whenever possible, and a nucleotide blast analysis was performed [12]. 

**Figure:**
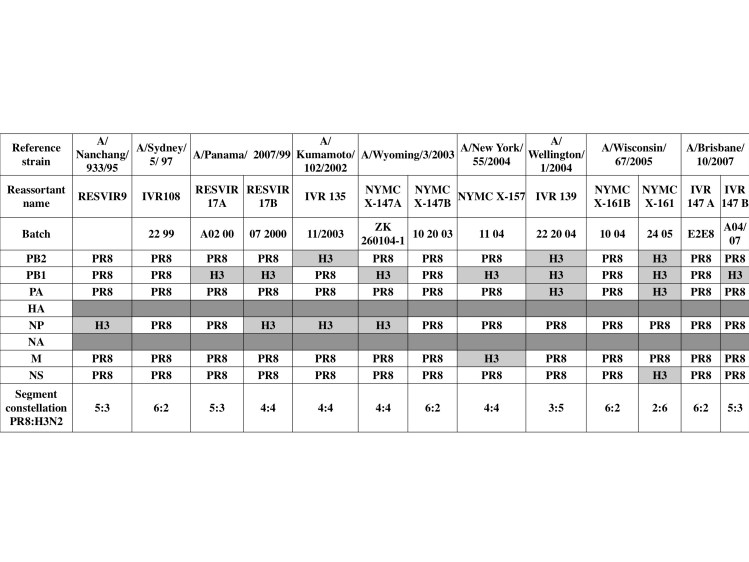

**Table 1. Gene constellation in vaccine seeds. **Viral RNAs of thirteen H3N2-based vaccine seeds were isolated directly from virus-containing allantoic fluid. After a RT step, specific segment PCRs were performed. All PCR products were purified and sequenced to determine the viral signature, i.e. PR8 or reference H3N2 strains. While vaccine seed selection is based on the presence of reference strains antigens, i.e. HA and NA, analysis of the two glycoprotein-encoding segments was not performed.

Coinfection byPR8 and the virus strains: A/Panama/2007/99, A/Wyoming/3/2003, A/Wisconsin/67/2005 and A/Brisbane/10/2007, generated two different reassortant viruses. Four vaccine seeds presented a 6:2 segment constellation (IVR108, NYMC X-147B, NYMC X-161B and IVR147 A). The nine remaining strains were a mosaic of internal PR8/H3N2 segments, ranging from one to four H3N2 segments completed by PR8 ones. No vaccine seeds, among the thirteen tested, contained more than four H3N2 internal segments.  One internal segment from H3N2 virus (NP or PB1), i.e. 5:3 reassortant, was incorporated in three vaccines seeds, RESVIR9, RESVIR17A and IVR147 B. Four vaccine seeds corresponded to 4:4 reassortant viruses (Table 2). They had incorporated two H3N2 internal segments: PB1 and NP (RESVIR17B and NYMC X-147A), PB2 and NP (IVR135) or PB1 and M segments (X-157). Two vaccine seeds presented the complete polymerase complex from H3N2 (the 3:5 reassortant IVR139 and the 2:6 reassortant NYMC X-161). The H3N2 PA segment was always incorporated with both H3N2 PB2 and PB1 segment. These encoded proteins for a homologous polymerase complex.

The majority of segments originated from PR8, but internal segments of reference H3N2 viruses were also incorporated (Figure 1). Overall, 54 % of the vaccine seeds (7 out of 13) contained   the H3N2 PB1 segment, which was thus the  H3N2 segment with highest incorporation rate. H3N2 NP segment was present in 4 out of 13 (31%) of the vaccine seeds. H3N2 PB2 and PA segments represented respectively 23% and 15% of the total segments. Finally, H3N2 M and NS segments were detected once in the thirteen seeds analyzed (8%).

**Figure:**
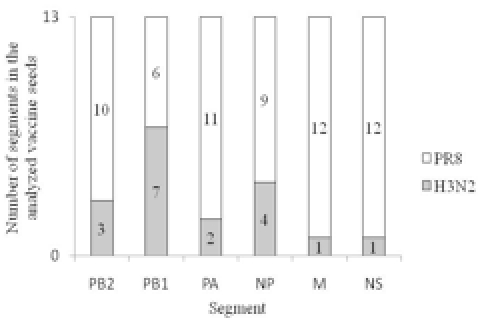

**Figure 1. Distribution of each segment according to their viral origin PR8 or H3N2 reference strain in the thirteen analyzed vaccine seeds**.

Aside from segment determination, reassortant viruses presented five different PR8:H3N2 gene constellations (Table 2). All internal H3N2 segments were incorporated at least once in vaccine seeds. 30 % of the tested vaccine seeds (4 out of 13) contained a 6:2 gene composition. One quarter of vaccine seeds presented a 5:3 constellation. About one-third of analyzed seeds incorporated two H3N2 internal segments, leading to 4:4 reassortants. One seed (IVR139) presented a 3:5 constellation and one another (NYMC X-161) a 2:6 constellation. 

**Figure:**
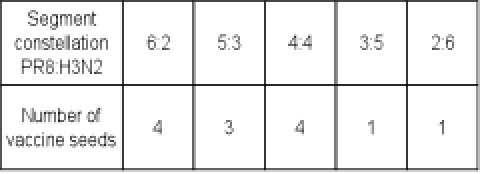

** Table 2. Distribution of vaccine seeds according to their genetic composition.**

## **Discussion**

Reassortment events in influenza viruses occur in a non -random way [14] due to associations between putative segments during packaging [15] and functional cooperation of proteins [16] . Influenza H3N2 vaccine seeds produced by co-infection were never entirely determined. In recent years, manufacturers have begun to characterize some of the internal segments, i.e. NP and M segments, to ensure that they contain a PR8 origin. The number of segments corresponding to PR8 segments in the vaccine seed seems to define the pathogenicity [17] and the replicative capacities of the virus. We sequenced thirteen H3N2 vaccine seeds to define their genetic composition. Incorporation of H3N2 internal segment(s) in vaccine seeds has not been reported before in spite of work based on reassortment events between PR8 and H3N2 viruses [14] [18]. Numerous studies considered that influenza vaccine seeds corresponded to 6:2 reassortants, i.e. PR8 genetic background completed with reference strain glycoproteins-encoding segments [[5], [19] [20] [21] ] . We demonstrate here that two thirds of vaccine seeds contained a mosaic of internal segments from the two viral origins, PR8 and H3N2 reference strain. Internal H3N2 segments were not incorporated with the same rate even if all of them have been packaged at least once. H3N2 M segment was encountered only once in analyzed vaccine seeds, despite the association of M proteins with others viral constituents [16] [22] [23] and compatibility degree described between HA and M segments [ [24] . However, our results are in agreement with the study of Lubeck *et al.* showing a preferential incorporation of PR8 M segment in high yields reassortant viruses from co-infection experiments between PR8 and H3N2 viruses in cell lines [14]. Interestingly, the H3N2 PA segment was incorporated only in association with H3N2 PB2 and PB1 segments suggesting a role of PA in the polymerase complex stability [25] [26] or in nuclear transport of the polymerase subunits [27] . 

Two vaccine seeds have been produced for four reference strains. The main reason for both of them (A/Wyoming/3/2003 (H3N2) and A/Wisconsin/67/2005 (H3N2)) being an inefficient production of the first reassortant (manufacturer’s data). The difference in the gene constellation could explain differences in replication. In this study, we show that the genetic content of vaccine seeds is a mosaic of parental strains. PB1 segment is the most representative H3N2 segment incorporated in analyzed vaccine seeds. The presence of H3N2 PB1 segment in vaccines as well as in the 2009 H1N1 pandemic influenza virus must provide a selective advantage that needs further investigation.  

## **Acknowledgements**

Thanks to Sylvaine Faure for technical resources and to Jean-Sébastien Casalegno.

## **Funding sources**

CB was funded by the Ministry of Education and Research, France.

## **Competing interests**

The authors declare that no competing interests exist.
